# The physiological, perceptual and neuromuscular responses of team sport athletes to a running and cycling high intensity interval training session

**DOI:** 10.1007/s00421-022-05053-8

**Published:** 2022-10-07

**Authors:** Craig Twist, Richard Bott, Jamie Highton

**Affiliations:** Department of Sport and Exercise Science, University of Chester, Parkgate Road, Chester, CH1 4BJ England, UK

**Keywords:** Exercise mode, Cardiorespiratory, Metabolic, Muscle function, Adaptation

## Abstract

**Purpose:**

The acute physiological, perceptual and neuromuscular responses to volume-matched running and cycling high intensity interval training (HIIT) were studied in team sport athletes.

**Methods:**

In a randomized cross-over design, 11 male team sport players completed 3 × 6 min (with 5 min between sets) repeated efforts of 15 s exercising at 120% speed (s$\dot{\text{V}}$O_2max_) or power (p$\dot{\text{V}}$O_2max_) at $\dot{\text{V}}$O_2max_ followed by 15 s passive recovery on a treadmill or cycle ergometer, respectively.

**Results:**

Absolute mean $\dot{\text{V}}$O_2_ (ES [95% CI] = 1.46 [0.47–2.34], *p* < 0.001) and heart rate (ES [95% CI] = 1.53 [0.53–2.41], *p* = 0.001) were higher in running than cycling HIIT. Total time at > 90% $\dot{\text{V}}$O_2max_ during the HIIT was higher for running compared to cycling (ES [95% CI] = 1.21 [0.26–2.07], *p* = 0.015). Overall differential RPE (dRPE) (ES [95% CI] = 0.55 [− 0.32–1.38], *p* = 0.094) and legs dRPE (ES [95% CI] = − 0.65 [− 1.48–0.23], *p* = 0.111) were similar, whereas breathing dRPE (ES [95% CI] = 1.01 [0.08–1.85], *p* = 0.012) was higher for running. Maximal isometric knee extension force was unchanged after running (ES [95% CI] = − 0.04 [− 0.80–0.8], *p* = 0.726) compared to a moderate reduction after cycling (ES [95% CI] = − 1.17 [− 2.02–0.22], *p* = 0.001).

**Conclusion:**

Cycling HIIT in team sport athletes is unlikely to meet the requirements for improving run-specific metabolic adaptation but might offer a greater lower limb neuromuscular load.

## Introduction

As an appropriate stimulus for improving $\dot{\text{V}}$O_2max_, high intensity interval training (HIIT) offers a training approach that is easily accommodated into an individual’s training schedule to acutely or progressively overload the cardiopulmonary, metabolic and neuromuscular systems (Buchheit and Laursen 2013a, b; Dolci et al. 2020). Despite many studies examining the acute and chronic adaptive responses to HIIT in athletes (e.g., Dupont et al. 2002; Buchheit et al. 2009; Wong et al. 2010; Jones et al. 2015; Beard et al. 2019), little is known about how the selected training modality (i.e., running vs. cycling) influences an individual’s response to HIIT.

In many team sports, practitioners and rehabilitation staff will choose training practices that simultaneously reduce musculoskeletal load while promoting appropriate central and peripheral stimuli. The use of cycle-based HIIT has been used with team sport athletes over periods of 2–6 weeks to improve intermittent running and cycling performance (Jones et al. 2015; Hamlin et al. 2017; Beard et al. 2019; Thom et al. 2020), while others have reported no effect of 5 weeks of cycle-based HIIT on running performance (Goods et al. 2015). Cycling might be adopted for athletes who require more careful load management, e.g., during rehabilitation after injury or in the days after match play, or to provide alternative training stimuli (Mallol et al. 2020; Thom et al. 2020). However, the application of cycle-based training with team sport athletes seems to have been applied without any direct comparison of the physiological and neuromuscular responses when compared to that from similar running-based HIIT approaches.

Running and cycling at the same relative intensity evoke distinct physiological and perceptual responses that suggest different stimuli could be applied when adopted by the same athlete (Carter et al. 2000; Hill et al. 2003; Millet et al. 2009; Mclaren et al. 2016). For example, compared to cycling, running is performed at a higher absolute metabolic state with a much faster oxygen uptake kinetic response (Carter et al. 2000; Hill et al. 2003). Exercise modality also alters localized perceived effort, with running eliciting higher central and cycling higher peripheral sensations (Mclaren et al. 2016; Rampinini et al. 2016). Differences in loading have also been used to suggest task-dependent neuromuscular responses for HIIT protocols using repeated sprint running and cycling (Rampinini et al. 2016; Tomazin et al. 2017). However, the extent to which these muscle responses are observed after lower intensity HIIT remains unclear. Describing how those athletes who regularly perform running respond to cycling activity of the same format would offer valuable insight for those seeking to better understand the application of HIIT in team sport athletes. Therefore, using a perimaximal HIIT format (~ 120% $\dot{\text{V}}$O_2max_) that has been reported previously (15 s active: 15 s rest; Dupont et al. 2002; Buchheit et al. 2009), we sought to compare the acute metabolic, cardiovascular, perceptual and neuromuscular responses of team sport athletes to volume-matched running and cycling HIIT sessions. It was hypothesized that the relative metabolic and cardiovascular demand would be higher for running HIIT compared to cycling, despite a smaller reduction in muscle force. It was also hypothesized that perceptual responses using the differential RPE would be sensitive to the physiological and neuromuscular inputs of the exercise mode.

## Methods

With Department of Sport and Exercise Sciences ethics approval, 11 male university standard team sport players (age 20.0 ± 0.8 y, stature 181 ± 5 cm, body mass 82.3 ± 12.4 kg) participated in this study after providing written informed consent. Participants represented a range of team sports, including soccer, rugby and basketball. An a priori sample size calculation using G*Power 3.1.9.6 (Faul et al. 2007) informed our sample recruitment. A sample size of nine was estimated to detect a one-tailed effect of *d* = 0.93 with a power of 80% and error rate of 5% using a paired-samples *t* test. The effect size of interest (*d* = 0.93) was estimated based on differences in maximal oxygen uptake for running and cycling in trained students (McArdle and Magel 1970), with the one-tailed option selected because of the systematically higher $\dot{\text{V}}$O_2_ values observed in running compared to cycling (Millet et al, 2009). Our choice of power and error rate were based on common practice (Lakens 2022), but were ultimately arbitrary. Twelve participants were initially recruited to account for participant drop out. Additional participants began testing before 9 complete sets of data had been collected—it was decided it was appropriate to complete the data collection for these additional participants. Participants first attended the laboratory on two separate occasions (temperature: 20.5 ± 1.1 compared to (cf.) 20.3 ± 1.1 °C, *p* = 0.518; humidity: 52.8 ± 3.3 cf. 52.1 ± 4.0%, *p* = 0.623; Pbar: 763 ± 11 cf. 764 ± 11 mmHg, *p* = 0.902; all ES < 0.2) completing incremental tests to exhaustion to establish speed (s$\dot{\text{V}}$O_2max_) and power (p$\dot{\text{V}}$O_2max_) corresponding to maximal oxygen uptake ($\dot{\text{V}}$O_2max_) during running (H/P Cosmos, Pulsar, Nussdorf-Traunstein, Germany) and cycling (Lode Excalibur Sport, Lode Medical Technology, Groningen, The Netherlands), respectively. The protocols started at 100 W (cycling) or 8 km h^−1^ with a 1% incline (running) and increased by 20 W min^−1^ (cycling) and speed by 0.5 km h^−1^ min^−1^ (running) until volitional exhaustion. Volitional exhaustion was defined as either the point at which participants could no longer maintain a cycling cadence of 50 rev min^−1^, or the speed of the treadmill. Expired air was collected continuously throughout each exhaustive trial using a pre-calibrated metabolic cart (Quark RMR, Cosmed, Cosmed.S.R.L., Italy). Oxygen uptake ($\dot{\text{V}}$O_2_), was recorded breath-by-breath and later averaged over 30 s, with heart rate (HR) collected via telemetry (Garmin Premium HR, Garmin Ltd, Kansas, USA). $\dot{\text{V}}$O_2max_ was accepted as the highest $\dot{\text{V}}$O_2max_ averaged over 30 s.

Participants completed two HIIT trials using either running (H/P Cosmos, Pulsar, Nussdorf-Traunstein, Germany) or cycling (Lode Excalibur Sport, Lode Medical Technology, Groningen, The Netherlands) in a randomized cross-over design, with 5–7 days between trials (temperature: 20.6 ± 0.8 cf. 20.4 ± 0.7 ℃, *p* = 0.465; humidity 53.3 ± 5.0 cf. 54.7 ± 4.7%, *p* = 0.606; Pbar 763 ± 1.7 cf. 763 ± 4.4 mmHg, *p* = 0.872; all ES < 0.2). Each HIIT session comprised 15 s at 120% s$\dot{\text{V}}$O_2max_ (running; 15.9 ± 1.7 km h^−1^) or p$\dot{\text{V}}$O_2max_ (cycling; 301 ± 28 W) followed by 15 s passive recovery, repeated for 6 min. Participants completed 3 sets with a 5 min passive recovery between each 6 min bout. Passive recovery during running was achieved by the participants placing their hands on the handrails and straddling the treadmill belt, while during cycling, the participants remained seated and legs were stationary. During treadmill running, participants wore a safety harness. Oxygen uptake and heart rate were measured throughout, with values for mean $\dot{\text{V}}$O_2_ (absolute and relative to mode-specific maximum), time > 90% maximum values and energy expenditure (kcal min^−1^) calculated (Weir 1949). Blood lactate concentration (Lactate Pro II, Arkray, Japan) was recorded immediately after with differential rating of perceived exertion (dRPE) for overall exertion (dRPE-O), breathlessness (dRPE-B) and leg-muscle exertion (dRPE-L) recorded 30 min after each HIIT trial using the centiMax scale (CR100; Borg and Borg 2002). Maximal voluntary isometric contraction of the knee extensors (MVC) in the dominant limb was measured immediately before and after each HIIT trial (S Beam Load Cell, Richmond Industries, Reading, UK) with the participant seated and the knee angle fixed at 90 degrees.

### Statistical analysis

All comparisons are reported as effect sizes (Cohen’s d; mean difference between trials/pooled standard deviation) and 95% confidence intervals (ES [95% CI]), with threshold values of 0.0–0.2, trivial; 0.21–0.6, small; 0.61–1.2, moderate; 1.21–2.0, large; > 2.0, very large. These arbitrary thresholds were used in the absence of accepted minimum thresholds for changes in the measurements of interest. Effects with confidence intervals that crossed a small positive or negative change were classified as unclear. For those wishing to interpret the analysis using a more traditional approach, we provide *p* values based on appropriate null hypothesis tests, although any ES confidence interval that includes zero can be considered as *p* > 0.05. Data were checked for assumptions of normality using the Shapiro–Wilk test and were found to be normally distributed (*p* > 0.05). Differences in physiological and perceptual responses were analyzed using separate paired-samples *t *tests, whereas differences in time spent > 90%$\dot{\text{V}}$O_2max_ and %$\dot{\text{V}}$O_2max_ during each bout, and changes in MVC were examined using separate repeated-measures analysis of variance. All data were analyzed using SPSS (version 27, Chicago, Illinois, USA) or a custom-made spreadsheet (https://www.cem.org/effect-size-calculator).

## Results

s$\dot{\text{V}}$O_2max_ and p$\dot{\text{V}}$O_2max_ were 13.2 ± 1.4 km h^−1^ and 251 ± 23 W, respectively. There were moderate differences in $\dot{\text{V}}$O_2max_ between running and cycling for both relative (ES [95% CI] = 0.91 [0.0–1.75], *p* = 0.0017) and absolute values (ES [95% CI] = 0.9 [− 0.1–1.73], *p* = 0.0037). However, small differences were observed in HR maximum (ES [95% CI] = 0.44 [− 0.46–1.23], *p* = 0.0531), while B[La]_max_ was unclear between running and cycling maximum tests (ES [95% CI] = − 0.43 [− 1.26–0.43], *p* = 0.2985). Data for running and cycling maximal tests are shown in Table 1.

**Table 1:** Physiological responses to maximal running and cycling tests. Data are mean ± SD

	Running	Cycling
$\dot{\text{V}}$O_2max_ (ml kg^−1^ min^−1^)	48.1 ± 6.5*	42.8 ± 5.0
$\dot{\text{V}}$O_2max_ (ml min^−1^)	3993.3 ± 523.1*	3571.8 ± 412.0
HR_max_ (b min^−1^)	200 ± 11	196 ± 9
B[La]_max_ (mmol L^−1^)	12.5 ± 4.4	14.2 ± 3.4

*denotes different to cycling value (*p* < 0.05)

**Fig. 1:**
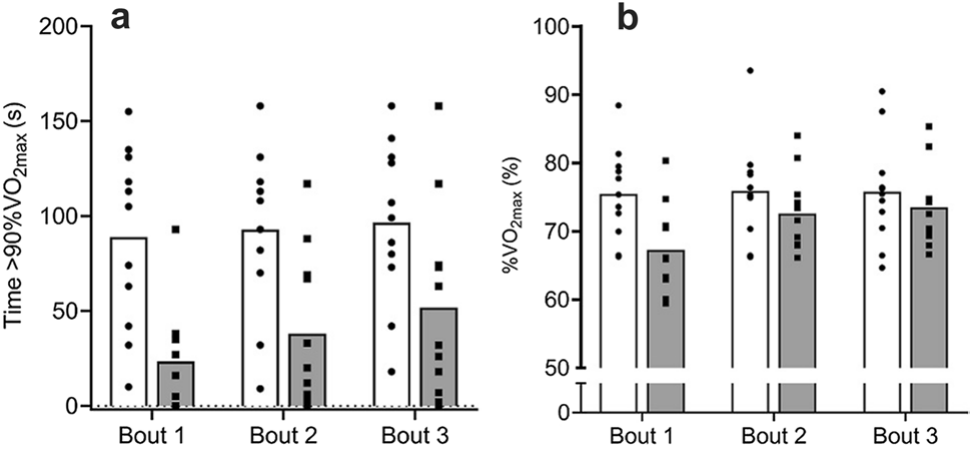
Time spent > 90%$\dot{\text{V}}$O_2max_ (**a**) and %$\dot{\text{V}}$O_2max_ (**b**) during each 3 × 6 min bout for running (closed circle) and cycling (closed square) HIIT. Values are mean (bars; white = running, grey = cycling) and individual responses

Physiological responses to running and cycling HIIT sessions are shown in Table 2. There was a large difference in absolute mean $\dot{\text{V}}$O_2_, with running HIIT higher than cycling (ES [95% CI] = 1.46 [0.47–2.34], *p* < 0.001), but not in $\dot{\text{V}}$O_2_ when considered as a proportion (%) of mode-specific $\dot{\text{V}}$O_2max_ (ES [95% CI] = 0.72 [− 0.17–1.55], *p* = 0.144). Total time > 90% $\dot{\text{V}}$O_2max_ during the HIIT was higher for running compared to cycling (ES [95% CI] = 1.21 [0.26–2.07], *p* = 0.015). Time spent > 90% $\dot{\text{V}}$ O_2max_ during running for bouts 1, 2 and 3 were (mean ± SD) 88.9 ± 47.4 s, 92.9 ± 43.1 s and 96.6 ± 42.6 s, respectively, with trivial differences between bout 1 and bout 2 (ES [95% CI] = − 0.09 [− 0.92–0.75], *p* = 0.549) and bout 2 and bout 3 (ES [95% CI] = − 0.09 [− 0.92–0.75], *p* = 0.709, Fig. 1a). For cycling, time spent > 90% $\dot{\text{V}}$O_2max_ was 23.4 ± 27.9 s, 38.0 ± 40.6 s and 51.8 ± 51.0 s for bouts 1–3, respectively, with small differences between bout 1 and bout 2 (ES [95% CI] = -0.42 [− 1.25–0.44], *p* < 0.01) and bout 2 and bout 3 (ES [95% CI] = − 0.30 [− 1.13–0.55], *p* < 0.01, Fig. 1a). The mean       %$\dot{\text{V}}$O_2max_ during running for bouts 1, 2 and 3 were 75.5 ± 6.6, 75.9 ± 7.4 and 75.8 ± 7.8%, respectively, with trivial differences between bout 1 and bout 2 (ES [95% CI] = − 0.06 [− 0.89–0.78], *p* = 0.549) and bout 2 and bout 3 (ES [95% CI] = 0.01 [− 0.82–0.85], *p* = 0.709, Fig. 1b). For cycling, the mean %$\dot{\text{V}}$O_2max_ during bouts 1, 2 and 3 were 67.3 ± 6.3, 72.6 ± 5.7 and 73.5 ± 5.8%, respectively, with small differences between bout 1 and bout 2 (ES [95% CI] = − 0.88 [− 1.72–0.02], *p* < 0.01) but trivial differences between bout 2 and bout 3 (ES [95% CI] = − 0.16 [− 0.99–0.69], *p* = 0.709, Fig. 1b).

**Table 2:** Physiological responses to running and cycling HIIT sessions

	Running	Cycling
Mean $\dot{\text{V}}$O_2_ (ml kg^−1^ min^−1^)	36.3 ± 4.7*	30.3 ± 3.4
Mean $\dot{\text{V}}$O_2_ (%VO_2max_)	75.7 ± 6.9	71.1 ± 5.9
Time > 90% $\dot{\text{V}}$O_2max_ (s)	288 ± 132*	128 ± 133
Mean $\dot{\text{V}}$E (L)	79.4 ± 9.4*	71.2 ± 5.7
Respiratory frequency (breath min^−1^)	41 ± 5*	35 ± 5
Mean HR (b min^−1^)	174 ± 12*	159 ± 7
Mean HR (%HR_max_)	87 ± 3*	81 ± 3
Time > 90% HR_max_ (s)	485 ± 255*	59 ± 110
B[La] (mmol L^−1^)	5.9 ± 2.5	4.7 ± 1.6
Energy expenditure (kcal min^−1^)	12.7 ± 1.2*	11.9 ± 1.2

*denotes different to cycling value (*p* < 0.05)

There were large differences in mean HR with running higher than cycling for absolute (ES [95% CI] = 1.53 [0.53–2.41], *p* = 0.001) and relative (% mode-specific maximum) values (ES [95% CI] = 2.00 [0.91–2.93], *p* = 0.002). There were also very large differences in total time > 90% HRmax with the running HIIT greater than cycling (ES [95% CI] = 2.17 [1.05–3.13], *p* < 0.001). Moderate differences in $\dot{\text{V}}$E (ES [95% CI] = 1.05 [0.13–1.90], *p* = 0.010) and large differences in respiratory frequency (ES [95% CI] = 1.30 [0.34–2.16], *p* = 0.020) also revealed higher values in running compared to cycling. Only small differences were observed in B[La] (ES [95% CI] = 0.57 [− 0.30–1.40], *p* = 0.054) and energy expenditure (ES [95% CI] = 0.67 [− 0.22–1.50], *p* = 0.008) after HIIT, with running higher than cycling. Data are shown in Table 2.

**Fig. 2:**
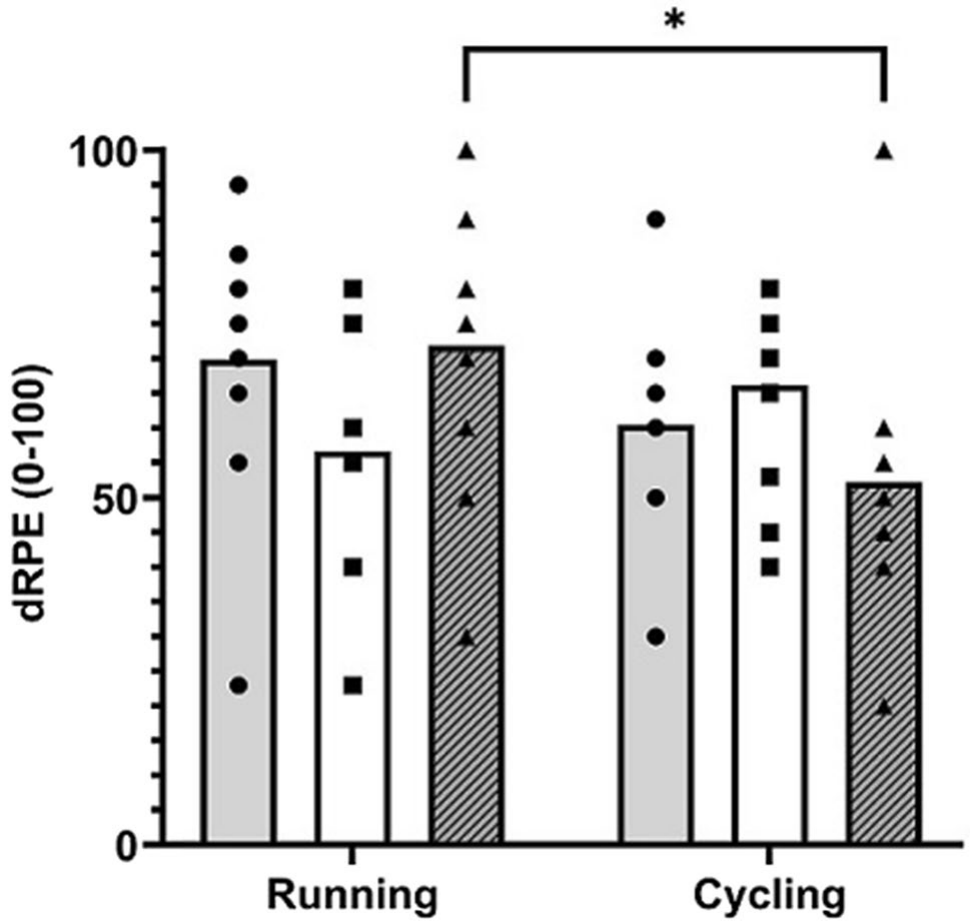
Differential rating of perceived exertion (dRPE) for overall exertion (dRPE-O; closed circle), leg-muscle exertion (dRPE-L; closed square) and breathlessness (dRPE-B; closed triangle) during running and cycling HIIT sessions. Values are mean (bars; overall = grey, leg = white, breathlessness = diagonal) and individual responses. *denotes difference between exercise modes (*p* < 0.05)

There were small differences in dRPE-O (69.8 ± 18.7 cf. 60.5 ± 14.7; ES [95% CI] = 0.55 [− 0.32–1.38], *p* = 0.094) and dRPE-L (56.6 ± 15.3 cf. 66.2 ± 14.2; ES [95% CI] = − 0.65 [− 1.48–0.23], *p* = 0.111) between running and cycling, respectively. However, dRPE-B (71.8 ± 19.3 cf. 52.3 ± 19.5; ES [95% CI] = 1.01 [0.08–1.85], *p* = 0.012) was moderately higher for running compared to cycling (Fig. 2).

**Fig. 3:**
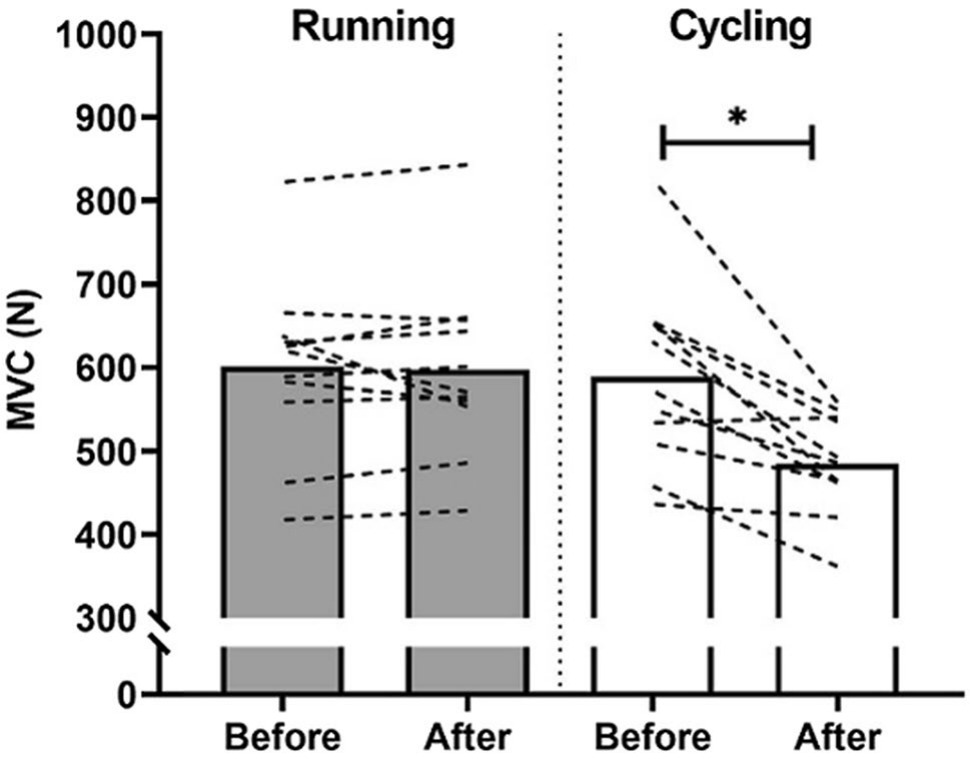
MVC before and after running and cycling-based HIIT sessions. Values are mean (bars; running = grey, cycling = white) and lines are individual responses. *Indicates different to before value (*p* < 0.05)

There was a trivial reduction in MVC (600.9 ± 105.6 to 597.0 ± 107.6 N; ∆% − 0.5 ± 5.8%) after running HIIT (ES [95% CI] = − 0.04 [− 0.80–0.8], *p* = 0.726) compared to a moderate reduction (588.5 ± 110–485.2 ± 59.9 N; ∆% − 16.3 ± 10.1%) after cycling HIIT (ES [95%CI] = − 1 .17 [− 2.02–0.22], *p* = 0.001). Data are shown in Fig. 3.

## Discussion

Running elicited a higher $\dot{\text{V}}$O_2max_ and HRmax for team sport athletes than cycling. A ~ 10% higher $\dot{\text{V}}$O_2max_ in running compared to cycling is attributed to the greater active muscle mass and the mode-specific adaptations that occur when running is a large proportion of the participant’s habitual training (Hill et al. 2003; Millet et al. 2009). While differences in HR_max_ are of a smaller magnitude (~ 2%) and less certain (i.e., with confidence intervals spanning a small decrease and increase), our observations are consistent with previous studies that report slightly lower HR values in cycling compared to running exercise (Roecker et al. 2003; Millet et al. 2009).

Given the greater capacity and requirement for oxygen consumption during running, absolute mean $\dot{\text{V}}$O_2_ during the HIIT running session was understandably higher than cycling. However, when mean $\dot{\text{V}}$ O_2_ during the HIIT session was expressed relative to mode-specific $\dot{\text{V}}$O_2max_, the difference between exercise modes was less clear—we observed a moderate difference, but our data were equally compatible with a large increase in running to a decrease with cycling. The relatively low overall oxygen demand for running (~ 76% $\dot{\text{V}}$O_2max_) and cycling (~ 70% $\dot{\text{V}}$O_2max_) during the 3 × 6 min bouts was noticeable, and similar to that reported before for studies adopting HIIT sessions comprising 15 s work: 15 s recovery (Rozenek et al. 2007). Rozenek and colleagues (2007) had participants run at intensities lower than those used in our study (i.e., 100% s$\dot{\text{V}}$O_2max_) but also incorporated an active recovery at 50% s$\dot{\text{V}}$O_2max_. The use of a passive recovery interval is likely to have contributed to the low mean %$\dot{\text{V}}$O_2max_ observed in our study (Buchheit and Laursen 2013a) and is therefore an important consideration when planning HIIT sessions, more so when using stationary cycling as the mode of exercise. The proportion of $\dot{\text{V}}$O_2max_ used during the running is also lower than that reported by Buchheit et al. (2009), who showed that during intermittent shuttle runs comprising 15 s work at 120% s$\dot{\text{V}}$O_2max_: 15 s passive recovery, mean $\dot{\text{V}}$O_2_ was ~ 88% of peak value. The use of treadmill running in the current study, where participants jumped on and off the treadmill moving at the set speed, is in contrast to the shuttle running on an indoor surface used by Buchheit et al. (2009). Participants on the treadmill would not be subject to the accelerations and decelerations inherent in shuttle running that would increase the metabolic cost of exercise (Stevens et al. 2015). The mode of how running is performed, i.e., outdoor cf. treadmill running, is therefore an important consideration for practitioners. The mean time at > 90% sVO_2max_ during running in our study (288 ± 132 s) was similar to values reported by Dupont et al. (2002) using the same running protocol (323 ± 272 s), albeit the within group variability was lower in our study. Differences are probably explained by Dupont and colleagues’ participants running over ground from a stationary start to cover a fixed distance in 15 s, meaning the need to accelerate and time at the required running speed would have fluctuated more, compared to our participants who ran on a treadmill at the fixed speed during the 15 s (Dupont et al. 2002).

Time spent at > 90% $\dot{\text{V}}$O_2max_ is a key parameter for adaptation to HIIT, with target times of ~ 5–7 min of total exercise time proposed for team sport athletes (Buchheit and Laursen 2013b; Paquette et al. 2019; Dolci et al. 2020). Running elicited a greater time > 90% $\dot{\text{V}}$O_2max_ than cycling HIIT, which equated to more than twice the total exercise time (27 ± 12% cf. 12 ± 12% total training session for running and cycling, respectively). Our confidence intervals for this observation provide some certainty that the effect is to increase time > 90%$\dot{\text{V}}$O_2max_, albeit this effect could range from small to very large. More time above the pre-defined threshold for adaptation in running compared to cycling might be explained by running possessing a higher metabolic demand that leads to a larger and faster primary phase of the $\dot{\text{V}}$O_2_ response (Hill et al. 2003). Millet et al. (2003) also reported a positive association between the time constant (τ) of the primary phase of the $\dot{\text{V}}$O_2_ kinetics and time above > 90% $\dot{\text{V}}$O_2max_. Faster oxygen uptake kinetics during running would mean that during the repeated 15 s efforts, the oxygen demands were met much sooner than in cycling and over the 18 min exercise period running elicited an improved opportunity to increase   $\dot{\text{V}}$O_2_ above the threshold. Notably, some participants recorded no or very limited time above the defined threshold during cycling (Fig. 1a). The use of short intervals (i.e., ≤ 15 s) using cycling in team sport athletes might therefore offer little value in targeting adaptations in $\dot{\text{V}}$O_2max_. Future studies exploring the responses to longer intervals and other HIIT types (e.g., sprint interval, repeated sprints) during off-feet training are needed.

While understanding the $\dot{\text{V}}$O_2_ response is an important measure of determining adaptation to HIIT, other measures of exercise intensity are typically used in practice. Mean HR was higher for running compared to cycling (Millet et al. 2009) and the time spent at > 90%HRmax was consistent with a moderate-to-very large difference between exercise modes. Albeit our selection of 90% of maximum values is arbitrary and might not represent the same physiological intensity (Achten and Jeukendrup 2003), the data suggest that in short intervals, such as those used here, heart rate is slower to respond in cycling compared to $\dot{\text{V}}$O_2_ (Midgley et al. 2007). This ‘lag’ in heart rate during short intervals reaffirms the challenges of using this measure to monitor exercise intensity and load during short-duration HIIT in team sport athletes (Buchheit et al. 2009).

The higher reported breathlessness (dRPE-B) for running probably reflected the higher metabolic demand and central responses (e.g., oxygen uptake, breathing rate, etc.) of this exercise modality, albeit our data were consistent with this effect being trivial to very large. Differences in favour of a higher rating of leg-exertion (dRPE-L) for cycling were more certain (small to large effects), and accompanied a greater reduction in MVC after this mode of exercise with similar certainty (small to large). Our use of dRPE therefore offered a potentially sensitive measure capable of differentiating between the specific central and peripheral inputs during HIIT (Mclaren et al. 2016).

Understanding the neuromuscular response to short-duration HIIT is necessary because of the potential impact on subsequent training sessions (Leveritt and Abernethy 1999) and that very little data examining muscle force after HIIT exercise exists. The cycling HIIT session caused moderate reductions (~ 16%) in MVC that were not observed after running HIIT (~ 1%). Indeed, reductions in MVC after running were unclear, in that our data were consistent with a moderate reduction and a moderate increase. If our observation of a trivial difference is correct, there are several potential mechanisms that could work either in isolation or combination to explain a greater force loss in cycling. A greater eccentric loading would be anticipated in running compared to cycling due to activation of the stretch–shortening cycle. Therefore, in running, force production would possibly have been enhanced for a given neural input (de Haan et al. 1991) delaying the onset of peripheral fatigue with a lower recruitment of type II motor units during running compared with cycling HIIT. Muscle activation across a number of muscle groups (i.e., knee extensors, knee flexors and plantar flexors) is likely to have occurred in running, whereas concentric actions of the knee extensors would predominate in cycling leading to a lower efficiency (Bijker et al. 2002). A greater contribution of the upper body musculature to overall $\dot{\text{V}}$O_2_ during running means the metabolic cost of upper body exercise during cycling makes a smaller contribution to the total exercise $\dot{\text{V}}$O_2_ . Therefore, in cycling HIIT, the lower body is more likely to be closer to its individual maximal oxygen consumption and its maximal voluntary contraction (Carter et al. 2000). This might require a progressive recruitment of the less efficient type II muscle fibres as the initially recruited type I fibres become fatigued, particularly given the exercising muscle is the principal origin of the slow component (Burnley and Jones 2007). During heavy cycling, there is high intramuscular tension and the recruitment of type II motor units is closely related to the requirements for muscle force generation (Carter et al. 2000). Higher intramuscular pressures might also cause partial occlusion of femoral arterial blood flow, reducing oxygen delivery that increases type II motor unit recruitment (Carter et al. 2000). While mechanisms remain speculative, we identify that cycling HIIT in team sport players resulted in a greater decrease in muscle force immediately after exercise than did running. This has clear implications for team sports that might use cycling as part of concurrent training practices. Further work exploring the recovery of muscle force after cycling HIIT in team sport athletes and impact on subsequent training is needed.

The study is not without limitations. First, the use of male university standard team sport athletes means that our findings might not translate directly to those athletes of a higher or lower standard or to female participants. Given the task-dependent nature of fatigue (Enoka and Duchateau 2008), we were unable to ascertain the central and peripheral components that contributed to the changes muscle force after cycling and if these differed to running. We also did not establish the cellular stress and molecular responses to HIIT for cycling and running. Finally, we acknowledge that our approach to sample size estimation has several limitations. Our anticipated effect size was estimated from a single study, which might have overestimated the population effect size. We selected a one-tailed test owing the typically higher $\dot{\text{V}}$O_2_ associated with running, but accept that we could not be certain of the direction of all effects, and therefore, a two-tailed option might have been more appropriate. Studies with sufficient resources might also wish to base their sample on a greater power than the commonly used 80% that we adopted. Indeed, a larger and more powerful sample would likely have improved the precision of our population estimates, which often had wide confidence intervals.

## Conclusion

This study examines the responses to a specific HIIT training session using cycle ergometry and running in team sport athletes, offering valuable insight to those team sport practitioners using cycle-based training with their athletes. These data highlight cycling elicited lower responses compared to running during short-duration (15 s) high intensity interval training. The time above the threshold for adaptation (i.e., > 90% $\dot{\text{V}}$O_2max_) is less likely to be met when using off-feet cycle training in team sport athletes. Short-interval cycling might offer a greater lower limb neuromuscular load without the need for accelerations, decelerations and changes of direction that are observed in running. When using cycling-based HIIT in team sport athletes where a high fractional utilization of maximum oxygen uptake is required, practitioners should also consider a prior warm-up that speeds the oxygen response (e.g., Jones et al. 2003) and the appropriateness of the work and rest interval intensity to elicit the required physiological stimulus.

## Abbreviations

B[La]: Blood lactate concentration
cf.: Confer (Latin), meaning ‘compared to’
dRPE: Differential rating of perceived exertion
ES: Effect size
HIIT: High intensity interval training
HR: Heart rate
MVC: Maximal voluntary isometric contraction
Pbar: Barometric pressure
$\dot{\text{V}}$E: Volume of expired air
p$\dot{\text{V}}$O2max: Power at maximum oxygen uptake
s$\dot{\text{V}}$O2max: Speed at maximum oxygen uptake
$\dot{\text{V}}$O2max: Maximum oxygen uptake
$\dot{\text{V}}$O2: Volume of oxygen
